# Activity of Colistin in Combination with Meropenem, Tigecycline, Fosfomycin, Fusidic Acid, Rifampin or Sulbactam against Extensively Drug-Resistant *Acinetobacter baumannii* in a Murine Thigh-Infection Model

**DOI:** 10.1371/journal.pone.0157757

**Published:** 2016-06-17

**Authors:** Bing Fan, Jie Guan, Xiumei Wang, Yulong Cong

**Affiliations:** 1 Clinical Laboratory of South Building, Chinese People’s Liberation Army General Hospital, Beijing 100853, China; 2 Clinical Laboratory of the Second Clinical District, the General Hospital of Chinese People’s Armed Police Forces, Beijing 100039, China; 3 Department of Clinical Laboratory, Peking University First Hospital, Beijing 100034, China; 4 Department of Clinical Laboratory, the General Hospital of Chinese People’s Armed Police Forces, Beijing 100039, China; University of Cape Town, SOUTH AFRICA

## Abstract

Few effective therapeutic options are available for treating severe infections caused by extensively drug-resistant *Acinetobacter baumannii* (XDR-AB). Using a murine thigh-infection model, we examined the *in vivo* efficacy of colistin in combination with meropenem, tigecycline, fosfomycin, fusidic acid, rifampin, or sulbactam against 12 XDR-AB strains. Colistin, tigecycline, rifampin, and sulbactam monotherapy significantly decreased bacterial counts in murine thigh infections compared with those observed in control mice receiving no treatment. Colistin was the most effective agent tested, displaying bactericidal activity against 91.7% of strains at 48 h post-treatment. With strains showing a relatively low minimum inhibitory concentration (MIC) for meropenem (MIC ≤ 32 mg/L), combination therapy with colistin plus meropenem caused synergistic inhibition at both 24 h and 48 h post-treatment. However, when the meropenem MIC was ≥64 mg/L, meropenem did not significantly alter the efficacy of colistin. The addition of rifampin and fusidic acid significantly improved the efficacy of colistin, showing a synergistic effect in 100% and 58.3% of strains after 24 h of treatment, respectively, while the addition of tigecycline, fosfomycin, or sulbactam did not show obvious synergistic activity. No clear differences in activities were observed between colistin-rifampin and colistin-fusidic acid combination therapy with most strains. Overall, our *in vivo* study showed that administering colistin in combination with rifampin or fusidic acid is more efficacious in treating XDR-AB infections than other combinations. The colistin-meropenem combination may be another appropriate option if the MIC is ≤32 mg/L. Further clinical studies are urgently needed to confirm the relevance of these findings.

## Introduction

*Acinetobacter baumannii* is a non-fermentative Gram-negative coccobacillus, whose natural reservoir remains to be determined [1]. It has emerged as one of the most significant nosocomial pathogens in health-care settings. Carbapenems were one of the most active agents against *A*. *baumannii*, but due to the overuse of these drugs, carbapenem-resistant strains have rapidly emerged during the last decade [2]. Most carbapenem-resistant *A*. *baumannii* are not only resistant to carbapenems, but are also highly resistant to nearly all commonly used antibiotic classes in clinical use; such strains are referred to as extensively drug-resistant *A*. *baumannii* (XDR-AB) [3]. Because of the limited number of effective therapeutic options available, severe infections caused by XDR-AB are often associated with high treatment failures and mortality rates [4].

Colistin, an “old drug” that was abandoned in the 1960s because of it severe nephrotoxicity, has been reintroduced in clinical settings. It exhibits rapid and concentration-dependent bactericidal activity by destroying the outer membrane of Gram-negative bacteria [5]. Results from several *in vitro* susceptibility tests have demonstrated its robust bactericidal activity against XDR-AB [6]. However, observations of rapid regrowth following colistin treatment *in vitro*, heteroresistance, and low plasma concentrations have raised questions regarding the efficacy of colistin as a monotherapy [7]. Thus, many physicians prefer to prescribe combination therapy to treat XDR-AB infections, especially considering the synergistic effects observed between colistin and other antibiotics, which have been proven in various *in vitro* studies [8].

However, synergistic activity found in *in vitro* tests may not correlate well with *in vivo* outcomes [9]. In addition, specific combinations showing increased *in vivo* efficacy against XDR-AB have not been well investigated. Therefore, to establish the potential use of combination therapy with colistin in clinical situations, we developed a murine thigh-infection model and employed this model to examine the *in vivo* efficacy of colistin combined with meropenem, tigecycline, fosfomycin, fusidic acid, rifampin, or sulbactam against XDR-AB.

## Materials and Methods

### Bacterial strains and molecular analysis

Twelve XDR-AB strains were collected from hospitalized patients of a tertiary hospital between 2013 and 2014 in Beijing, China. Five strains were isolated from the sputa of patients with pneumonia, 4 strains were isolated from the blood, and the 3 remaining strains were isolated from urine. *Pseudomonas aeruginosa* ATCC27853 and *Escherichia coli* ATCC25922 were used as reference strains for susceptibility testing. All strains were identified using the Vitek® 2 Compact System (bioMérieux, Marcyl’Étoile, France).

Pulsed-field gel electrophoresis (PFGE) was performed on the tested strains. In brief, the strains were digested with proteinase K (Takara Biotechnology Co. LTD., Dalian, China), and chromosomal DNA was digested with ApaI (Takara Biotechnology Co. LTD.) as previously described [10]. The PFGE was run in a CHEFMapperTM system (Bio-Rad Laboratories, Inc., Berkeley, CA, USA), and the DNA band profiles were detected after staining with ethidium bromide and photographed with the UV gel Doc (BIO-RAD, USA). The similarity between isolates was determined by the comparison of the DNA banding patterns using the BioNumerics V 6.0 software (Bio-Rad) according to the criteria of Tenover et al. [11]. All isolates with PFGE banding patterns with a similarity index of >75% were grouped within the same cluster. Then the Ambler class B carbapenemase *bla*_*IMP-1*_, *bla*_*IMP-2*_, *bla*_*NDM-1*_, *bla*_*SIM-1*_, *bla*_*VIM-1*_ *and bla*_*VIM-2*_ and Ambler class D carbapenemase *bla*_*OXA-23*,_ *bla*_*OXA-24*,_ *bla*_*OXA-51*,_ *bla*_*OXA-58*_ were further detected by PCR as previously described [12]. The primers used in this study are shown in Table 1.

**Table 1:** PCR primers and product sizes for the detection of carbapenemases among 12 XDR-AB strains

Target gene	Primers	Product size (bp)
*bla*_*VIM-1*_	5’-GGGAGCCGAGTGGTGAGT-3’; 5’-GGCACAACCACCGTATAG-3’	519
*bla*_*VIM-2*_	5’-ATGTTCAAACTTTTGAGTAAG-3’; 5’-CTACTCAACGACTGAGCG-3’	801
*bla*_*IMP-1*_	5’-ACCGCAGCAGAGTCTTTGCC-3’; 5’-ACAACCAGTTTTGCCTTACC-3’	587
*bla*_*IMP-2*_	5’-GTTTTATGTGTATGCTTCC-3’; 5’-AGCCTGTTCCCATGTAC-3’	678
*bla*_*SIM-1*_	5’-TACAAGGGATTCGGCATCG-3’; 5’- TAATGGCCTGTTCCCATGTG-3’	571
*bla*_*NDM-1*_	5’-ATGGAATTGCCCAATATTATGCACCCGG-3’; 5’- TCAGCGCAGCTTGTCGGCCATG -3’	813
*bla*_*OXA-23*_	5’-GATCGGATTGGAGAACCAGA-3’; 5’-ATTTCTGACCGCATTTCCAT-3’	501
*bla*_*OXA-24*_	5’-GGTTAGTTGGCCCCCTTAAA-3’; 5’-AGTTGAGCGAAAAGGGGATT-3’	246
*bla*_*OXA-51*_	5’-TAATGCTTTGATCGGCCTTG-3’; 5’-TGGATTGCACTTCATCTTGG-3’	353
*bla*_*OXA-58*_	5’-AAGTATTGGGGCTTGTGCTG-3’; 5’-CCCCTCTGCGCTCTACATAC-3’	599

### Antibiotics

Meropenem, fosfomycin, fusidic acid, rifampin, and sulbactam standards were obtained from the National Institute for the Control of Pharmaceutical and Biological Products (Beijing, China). Tigecycline and colistin was purchased from Sigma-Aldrich (St Louis, MO, USA).

### Susceptibility testing

The minimum inhibitory concentration (MIC) of all antibiotics was determined using the broth-microdilution method, according to the Clinical and Laboratory Standard Institute (CLSI) guidelines [13,14]. Briefly, cation-adjusted Mueller-Hinton broth (Becton, Dickinson and Co.; Franklin Lakes, NJ, USA) containing graded concentrations of antibiotics was freshly prepared. Study isolates that grew to an optic density of 0.5 McFarland units were diluted to 5 ×10^6^ CFU/ml. Then 100 μl of antibiotic solution and 10 μl of bacteria suspension were added simultaneously to 96-well U-bottom microplates. After mixing with a vortexer, the microplates were incubated for 24 h at 37°C in ambient air. All susceptibility tests were performed in 3 independent experiments on different days.

### Mouse thigh-infection model

This animal study was approved by the Research Animal Care and Use Committee of the General Hospital of Chinese People’s Armed Police Forces (No.WJ27856). Specific-pathogen-free, female Bagg inbred albino c-strain (BALB/c) mice (Charles River, Beijing, China) weighing 25 ± 2 g were used in this study. Mice were maintained and utilized according to the Protocol for the Protection and Welfare of Animals. The thigh-infection model was constructed as described previously [15]. Before inoculation, mice were rendered neutropenic by intraperitoneal injection with cyclophosphamide (Bristol-Myers Squibb, Princeton, NJ) at 150 mg/kg body weight (4 days before infection) or 100 mg/kg (1 day before infection). To develop the thigh-infection model, 0.1 ml freshly prepared bacterial suspension at a density of 3 × 10^7^ CFU/ml was intramuscularly injected into the left thigh of each mouse. After inoculation, they were randomly divided into 3 groups (5 mice/group) receiving monotherapy, combination therapy, or no treatment for 24 h or 48 h observation (a total of 6 groups for each strain). The following antibiotic doses were used: colistin at 20 mg/(kg·8 h) [16], meropenem at 200 mg/(kg·8 h) [17], tigecycline at 50 mg/(kg·24 h) [18], fosfomycin at 100 mg/(kg·4 h) [19] and fusidic acid 500 mg/(kg·8 h) [20], rifampin at 25 mg/(kg·6 h) [16], and sulbactam 120 mg/(kg·12 h) [21], as described previously. At 24 h or 48 h post-treatment, the infected mice were humanely euthanized, and their thigh muscles were aseptically excised. The thigh muscles were then homogenized and the number of CFUs was counted after serially diluting the homogenates.

All statistical analyses were performed using IBM SPSS software (version 20.0). Data for each group were presented as the mean value, and a *t* test was used for statistical analysis. A treatment regimen was considered effective if it resulted in a statistically significant reduction of bacterial counts (*P* < 0.05) when compared with no treatment or other regimens. Synergy for a combination therapy was defined as a ≥2 log10 CFU/mL decrease in comparison with the single drug, and antagonism was defined as ≥2 log10 CFU/mL increase. A ≥3 log10 CFU/mL decrease was considered to indicate bactericidal levels of activity.

## Results

### Susceptibility testing and molecular analysis

**Fig 1:**
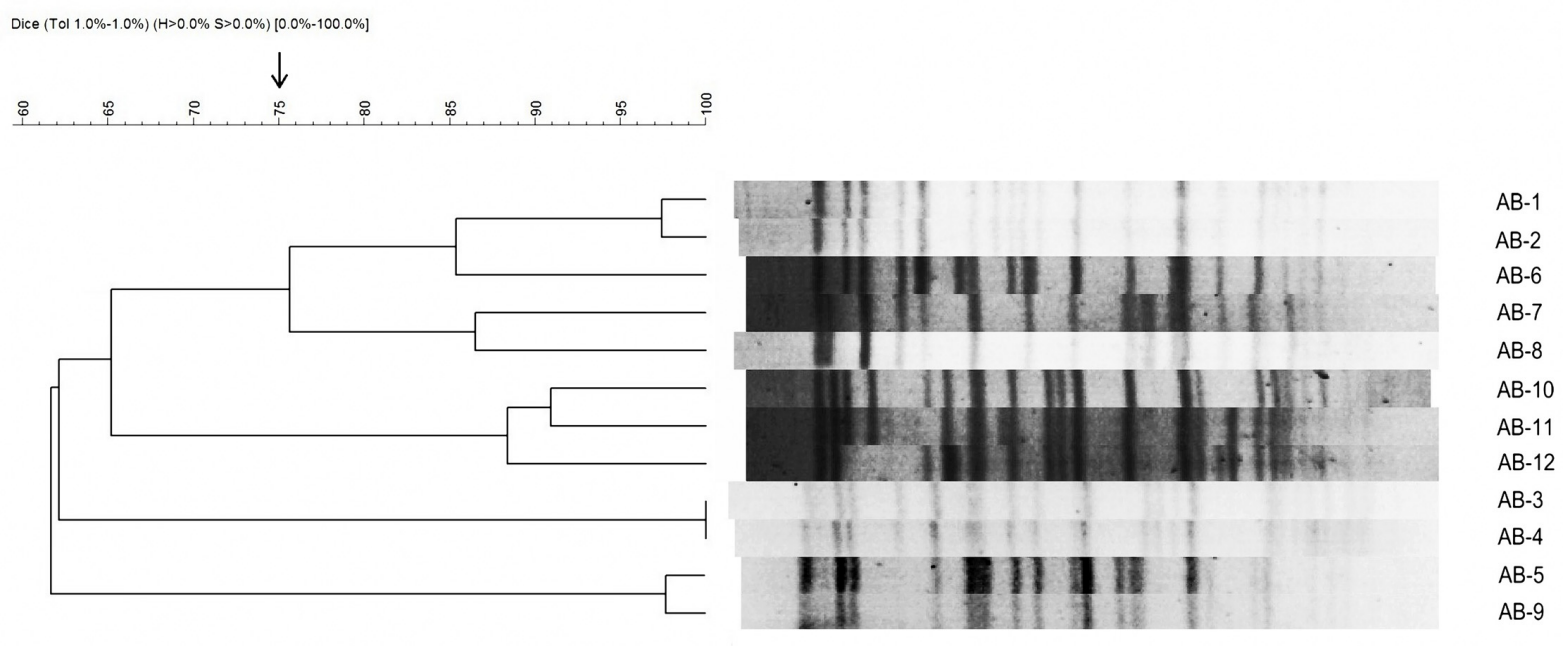
Pulsed-field gel electrophoresis analysis of genomic DNA from the 12 tested strains of *Acinetobacter baumannii*.

As shown in Fig 1, the PFGE patterns classified the 12 isolates into 4 distinct clonal types. The *bla*_*OXA-51*_ carbapenemase gene was detected in all strains, and *bla*_*OXA-23*_ was detected in 8 strains. Ambler class B carbapenemase genes were all negative. All strains were resistant to meropenem and susceptible to colistin, based on breakpoints determined using the CLSI guidelines (Table 2). The test strains also had low MICs for tigecycline (0.5 to 2 mg/L), but relatively high MICs for other antibiotics.

**Table 2:** Susceptibilities and molecular characteristics of 12 XDR-AB strains^a^.

	Clonal types	Carbapenemases	MIC (mg/L)						
Strain NO.			CST	MER	TIG	FOS	FD	RIF	SUL
AB-1	A	*bla*_*OXA-51*,_ *bla*_*OXA-23*_	0.5	32	1	>512	64	8	64
AB-2	A	*bla*_*OXA-51*_	0.5	32	1	>512	128	16	32
AB-3	B	*bla*_*OXA-51*,_ *bla*_*OXA-23*_	0.5	32	1	>512	64	16	64
AB-4	B	*bla*_*OXA-51*_	0.5	16	2	>512	32	64	128
AB-5	C	*bla*_*OXA-51*_	1	16	1	>512	128	16	32
AB-6	A	*bla*_*OXA-51*_	0.5	64	0.5	>512	16	32	64
AB-7	A	*bla*_*OXA-51*,_ *bla*_*OXA-23*_	0.5	64	1	>512	32	32	32
AB-8	A	*bla*_*OXA-51*,_ *bla*_*OXA-23*_	0.5	64	1	>512	64	64	32
AB-9	C	*bla*_*OXA-51*,_ *bla*_*OXA-23*_	0.25	128	0.5	>512	32	64	128
AB-10	D	*bla*_*OXA-51*,_ *bla*_*OXA-23*_	0.5	64	1	>512	32	128	16
AB-11	D	*bla*_*OXA-51*,_ *bla*_*OXA-23*_	0.5	64	2	>512	64	32	32
AB-12	D	*bla*_*OXA-51*,_ *bla*_*OXA-23*_	0.25	128	2	>512	256	64	64

^a^ CST, colistin; MER, meropenem; TIG, tigecycline; FOS, fosfomycin; FD, fusidic acid; RIF, rifampin; SUL, sulbactam.

### Efficacy of antibiotic monotherapies compared with no treatment

The efficacies of antibiotic monotherapies, which were reflected by decreased bacterial counts, are shown in Table 3. Colistin, tigecycline, rifampin, and sulbactam monotherapies showed statistically significant decreases of bacterial counts for all 12 strains at 24 h and 48 h post-treatment. Compared with other antibiotics, colistin had the highest efficacy and showed bactericidal activity (≥3 log10 CFU/mL decrease) with 91.7% (11/12) of strains after a 48-h treatment. Tigecycline and rifampin monotherapy displayed bactericidal activity in 50% (6/12) and 58.3% (7/12) of strains after 48 h of treatment, respectively. Monotherapy with meropenem, fosfomycin, and fusidic acid exhibited considerably lower activity, showing significant inhibition of 50% or less of the test strains after both 24 h and 48 h of treatment.

**Table 3:** Efficacies of antibiotic monotherapies after 24 and 48 h of treatment vs results for untreated animals^a^.

Strain NO.	CST VS no treatment		MER VS no treatment		TIG VS no treatment		FOS VS no treatment		FD VS no treatment		RIF VS no treatment		SUL VS no treatment	
	Δlogb^b^	*P* value^c^	Δlog	*P* value	Δlog	*P* value	Δlog	*P* value	Δlog	*P* value	Δlog	*P* value	Δlog	*P* value
**24 h of treatment**														
AB-1	-2.31	**<0.001**	-0.46	**<0.001**	-2.69	**<0.001**	-0.23	0.222	-0.18	**0.043**	-2.65	**<0.001**	-1.46	**<0.001**
AB-2	-2.22	**<0.001**	-0.01	0.574	-2.92	**<0.001**	-0.20	0.051	-0.24	**0.019**	-2.54	**<0.001**	-1.19	**<0.001**
AB-3	-2.14	**<0.001**	-0.15	**0.029**	-2.06	**<0.001**	-0.05	0.559	-0.19	**0.022**	-2.58	**<0.001**	-1.16	**<0.001**
AB-4	-1.68	**<0.001**	-0.92	**<0.001**	-1.54	**<0.001**	-0.01	0.919	-0.17	0.066	-2.55	**<0.001**	-0.67	**<0.001**
AB-5	-1.25	**<0.001**	-0.92	**0.001**	-2.88	**<0.001**	-0.20	**0.032**	-0.02	0.626	-2.68	**<0.001**	-1.56	**<0.001**
AB-6	-2.34	**<0.001**	0.04	0.367	-2.16	**<0.001**	-0.07	0.205	-0.36	**0.001**	-2.23	**<0.001**	-1.02	**<0.001**
AB-7	-2.63	**0.002**	0.09	0.259	-2.65	**0.002**	-0.01	0.867	-0.27	**0.026**	-3.18	**<0.001**	-1.26	**0.002**
AB-8	-2.46	**<0.001**	-0.15	**0.023**	-2.83	**<0.001**	-0.14	0.059	-0.12	0.100	-2.63	**<0.001**	-1.37	**<0.001**
AB-9	-2.59	**<0.001**	-0.25	**0.042**	-2.12	**<0.001**	0.08	0.068	-0.64	**<0.001**	-1.39	**<0.001**	-0.51	**<0.001**
AB-10	-2.48	**0.021**	-0.30	0.350	-2.11	**0.022**	-0.07	0.706	-0.34	0.226	-1.37	**0.024**	-2.54	**0.021**
AB-11	-2.06	**0.019**	-0.47	0.133	-1.28	**0.024**	-0.04	0.234	-0.44	0.754	-1.96	**0.019**	-1.44	**0.021**
AB-12	-2.27	**0.008**	-0.40	0.110	-0.89	**0.016**	0.01	0.123	-0.44	0.113	-1.45	**0.009**	-1.06	**0.012**
**48 h of treatment**														
AB-1	-3.05	**0.002**	0.09	0.241	-3.40	**0.002**	-0.10	0.296	-0.06	0.632	-2.38	**0.002**	-1.30	**<0.001**
AB-2	-3.12	**0.001**	0.04	0.598	-3.15	**0.001**	-0.08	0.563	-0.10	0.351	-3.20	**0.001**	-3.30	**0.001**
AB-3	-3.45	**<0.001**	0.00	0.913	-2.23	**<0.001**	-0.07	0.411	-0.15	0.064	-1.40	**<0.001**	-1.05	**0.002**
AB-4	-2.23	**0.002**	-0.03	0.697	-1.36	**0.003**	-0.14	0.173	0.16	0.142	-0.45	**0.012**	-1.84	**0.003**
AB-5	-3.28	**0.024**	-0.16	0.360	-3.31	**0.008**	-0.61	0.099	-0.53	0.657	-3.43	**0.008**	-2.03	**0.009**
AB-6	-3.26	**0.003**	-0.13	0.359	-2.59	**0.003**	-1.06	**0.005**	-1.09	**0.005**	-3.36	**0.003**	-2.37	**0.003**
AB-7	-3.35	**0.014**	-0.17	0.716	-3.44	**0.014**	-0.15	0.518	-0.28	0.829	-3.42	**0.014**	-1.88	**0.016**
AB-8	-3.29	**0.005**	-0.22	0.962	-3.38	**0.005**	-0.92	**0.009**	-0.15	0.719	-2.48	**0.005**	-3.29	**0.005**
AB-9	-3.42	**0.001**	-0.20	0.425	-2.55	**0.001**	-0.33	0.057	-0.15	0.857	-3.29	**0.001**	-2.26	**0.001**
AB-10	-3.26	**0.001**	-0.23	0.322	-3.28	**0.001**	-0.73	**0.001**	-0.03	0.222	-1.05	**0.002**	-3.37	**0.001**
AB-11	-3.35	**0.004**	-0.20	0.594	-1.51	**0.005**	-0.29	0.272	-0.12	0.640	-3.44	**0.004**	-3.43	**0.004**
AB-12	-3.04	**0.001**	-0.34	0.478	-1.43	**0.001**	-0.96	**0.002**	-0.32	0.858	-3.41	**0.001**	-2.28	**0.001**

^a^ CST, colistin; MER, meropenem; TIG, tigecycline; FOS, fosfomycin; FD, fusidic acid; RIF, rifampin; SUL, sulbactam.

^b^ Δlog means the log_10_ recovered CFU of antibiotic monotherapy minus the log_10_ recovered CFU of no treatment.

^c^ A *P* value of < 0.05 indicates significance and is shown in bold.

### Efficacy of colistin combinations compared with monotherapies

The efficacies of combination therapies with colistin were compared with monotherapies (Table 4). The addition of rifampin and fusidic acid significantly improved the efficacy of colistin, showing synergistic effects against 100% and 58.3% of strains after 24 h of treatment, respectively. With strains with relatively low meropenem MICs (AB-1 to AB-5), colistin plus meropenem administration displayed synergistic effects after both 24 h and 48 h of treatment. However, when the meropenem MIC was ≥64 mg/L (AB-6 to AB-12), meropenem addition did not significantly alter the efficacy of colistin. Though colistin plus fosfomycin treatment was more effective (*P* < 0.05) than colistin monotherapy in 83.3% (24 h) and 66.7% (48 h) of the test strains, synergistic inhibition was only found in 1 strain (AB-4). Combined administration of tigecycline or sulbactam with colistin did not considerably improve the efficacy of colistin. No antagonism was observed in this study.

**Table 4:** Comparison of the efficacies of colistin combinations vs monotherapies after 24 and 48 h of treatment^a^.

Strain NO.	CST+MER VS CST		CST+TIG VS CST		CST+TIG VS TIG		CST+FOS VS CST		CST+FD VS CST		CST+RIF VS CST		CST+RIF VS RIF		CST+SUL VS CST		CST+SUL VS SUL	
	Δlog^b^	*P* value^c^	Δlog	*P* value	Δlog	*P* value	Δlog	*P* value	Δlog	*P* value	Δlog	*P* value	Δlog	*P* value	Δlog	*P* value	Δlog	*P* value
**24h of treatment**																		
AB-1	-2.05	**0.006**	-0.94	**0.009**	-0.56	0.253	-1.14	**0.007**	-2.78	**0.006**	-2.95	**0.006**	-2.61	**0.009**	-0.08	0.955	-0.94	**0.013**
AB-2	-2.11	**0.005**	-0.91	**0.008**	-0.07	0.589	-1.16	**0.006**	-2.14	**0.005**	-2.96	**0.005**	-2.64	**0.015**	-0.08	0.861	-1.12	**0.007**
AB-3	-3.02	**<0.001**	0.08	0.162	0.00	0.448	-1.25	**<0.001**	-2.93	**<0.001**	-3.02	**<0.001**	-2.59	**0.004**	-0.32	**0.002**	-1.31	**0.006**
AB-4	-2.88	**0.007**	-1.26	**0.009**	-1.39	**0.008**	-2.06	**0.033**	-2.70	**0.007**	-3.34	**0.007**	-2.46	**0.005**	-0.43	**0.018**	-1.43	**0.009**
AB-5	-3.09	**0.010**	-1.47	**0.011**	0.16	0.335	-1.18	**0.012**	-2.70	**0.010**	-4.18	**0.010**	-2.74	**0.004**	-1.19	**0.012**	-0.87	**0.003**
AB-6	-0.91	**0.008**	0.16	0.169	-0.02	0.844	0.20	0.066	-1.84	**0.006**	-2.29	**0.006**	-2.40	**0.004**	0.03	0.951	-1.29	**<0.001**
AB-7	-0.46	**0.012**	-0.44	**0.014**	0.04	0.973	-0.65	**0.007**	-2.40	**0.003**	-2.41	**0.003**	-2.86	**0.021**	0.04	0.706	-1.32	**0.004**
AB-8	0.02	0.969	-0.54	0.053	-0.17	0.153	-0.95	**0.032**	-1.88	**0.025**	-2.71	**0.024**	-2.54	**0.010**	-0.18	0.225	-1.27	**0.005**
AB-9	-0.20	0.211	-0.42	0.105	-0.02	0.625	-0.73	0.063	-1.56	**0.041**	-3.11	**0.039**	-4.32	0.086	0.45	**0.047**	-1.63	**0.004**
AB-10	-0.17	0.096	-0.43	**0.028**	-0.80	0.071	-0.70	**0.013**	-1.75	**0.007**	-2.55	**0.007**	-3.47	**0.012**	0.36	**0.003**	0.42	0.760
AB-11	-0.44	0.078	-0.13	0.654	-0.91	0.052	-0.98	**0.019**	-2.08	**0.014**	-2.82	**0.014**	-2.92	**0.030**	-0.03	0.834	-0.65	**0.004**
AB-12	0.12	0.446	-0.67	**0.022**	-0.31	0.288	-1.03	**0.014**	-1.41	**0.012**	-2.90	**0.011**	-3.71	**0.001**	-0.16	0.196	-1.36	**0.001**
**48h of treatment**																		
AB-1	-2.02	**0.002**	-0.67	**0.009**	-0.31	0.399	-0.50	**0.012**	-2.87	**0.002**	-2.83	**0.002**	-3.49	**0.008**	-0.26	0.050	-2.00	**0.014**
AB-2	-2.04	**0.002**	-0.69	**0.004**	-0.66	**0.013**	-0.45	**0.003**	-2.63	**0.002**	-2.73	**0.002**	-2.65	**0.001**	-0.01	0.795	0.17	0.142
AB-3	-2.59	**0.020**	-0.47	0.056	-1.69	**0.002**	-0.54	**0.045**	-2.54	**0.019**	-2.78	**0.019**	-4.83	**0.007**	0.16	0.447	-2.04	**0.014**
AB-4	-2.93	**0.010**	-0.90	0.054	-1.77	**0.009**	-0.05	0.500	-4.04	**0.009**	-3.80	**0.009**	-5.58	**0.024**	-1.21	**0.002**	-1.60	**0.041**
AB-5	-2.35	**0.011**	-0.45	**0.027**	-0.42	**0.009**	-0.16	0.233	-2.91	**0.011**	-3.15	**0.011**	-3.01	**0.001**	0.02	0.997	-1.24	**<0.001**
AB-6	-0.11	0.985	-0.25	0.243	-0.92	**0.005**	0.12	0.150	-2.56	**0.001**	-2.95	**0.001**	-2.85	**0.005**	0.01	0.867	-0.88	**0.001**
AB-7	0.21	0.086	-0.40	**0.010**	-0.31	**0.017**	-0.33	**0.017**	-2.36	**0.004**	-2.94	**0.004**	-2.87	**0.009**	-0.02	0.993	-1.49	**0.032**
AB-8	0.30	0.053	-0.27	0.747	-0.18	0.989	-0.75	**0.008**	-1.89	**0.004**	-3.10	**0.004**	-3.91	**0.010**	-0.15	0.182	-0.16	0.096
AB-9	-0.23	0.117	-0.33	**0.029**	-1.21	**0.001**	-0.34	**0.015**	-1.44	**0.004**	-3.04	**0.004**	-3.17	**0.001**	-0.34	**0.017**	-1.50	**0.002**
AB-10	-0.28	0.110	-0.13	0.087	-0.11	0.474	-0.70	**0.003**	-2.95	**0.002**	-2.94	**0.002**	-5.15	**0.007**	-0.14	0.313	0.54	0.515
AB-11	-0.09	0.566	-0.12	0.860	-1.95	**0.002**	-0.71	**0.015**	-2.49	**0.008**	-2.96	**0.008**	-2.87	**0.004**	-0.07	0.629	0.01	0.905
AB-12	-0.04	0.870	0.12	0.535	-1.49	**0.007**	-0.25	0.109	-1.59	**0.017**	-3.34	**0.016**	-2.97	**<0.001**	-0.12	0.283	-0.88	**0.004**

^a^ CST, colistin; MER, meropenem; TIG, tigecycline; FOS, fosfomycin; FD, fusidic acid; RIF, rifampin; SUL, sulbactam.

^b^ Δlog means the log_10_ recovered CFU of antibiotic combination therapy minus the log_10_ recovered CFU of monotherapy.

^c^ A *P* value of < 0.05 indicates significance and is shown in bold.

### Comparison of colistin combinations

**Fig 2:**
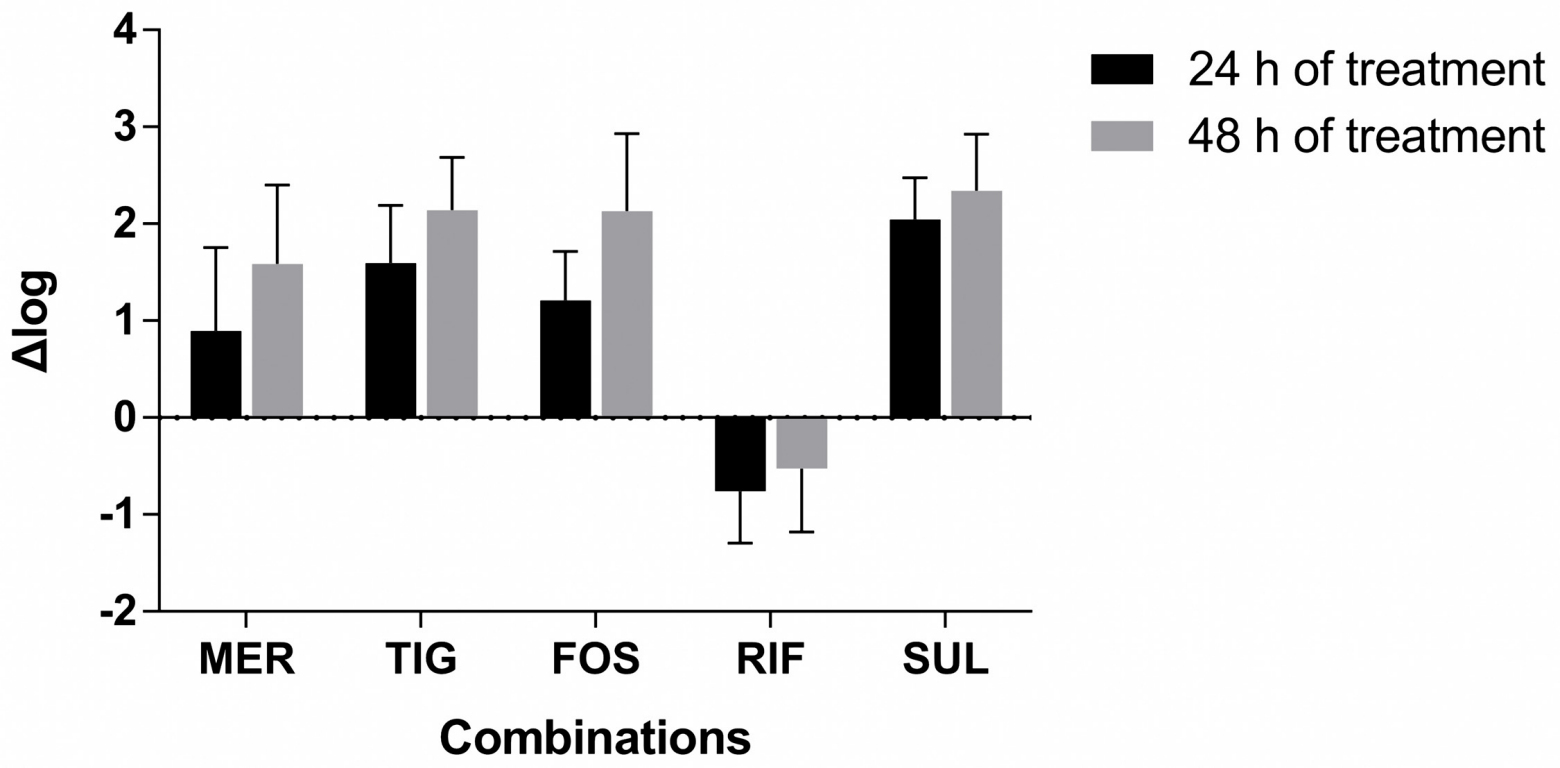
Comparison of the efficacies of colistin-fusidic acid combination vs other combinations after 24 and 48 h of treatment. CST, colistin; MER, meropenem; TIG, tigecycline; FOS, fosfomycin; FD, fusidic acid; RIF, rifampin; SUL, sulbactam. Δlog means the log_10_ recovered CFU of other combinations minus the log_10_ recovered CFU of colistin-fusidic acid combination.

**Fig 3:**
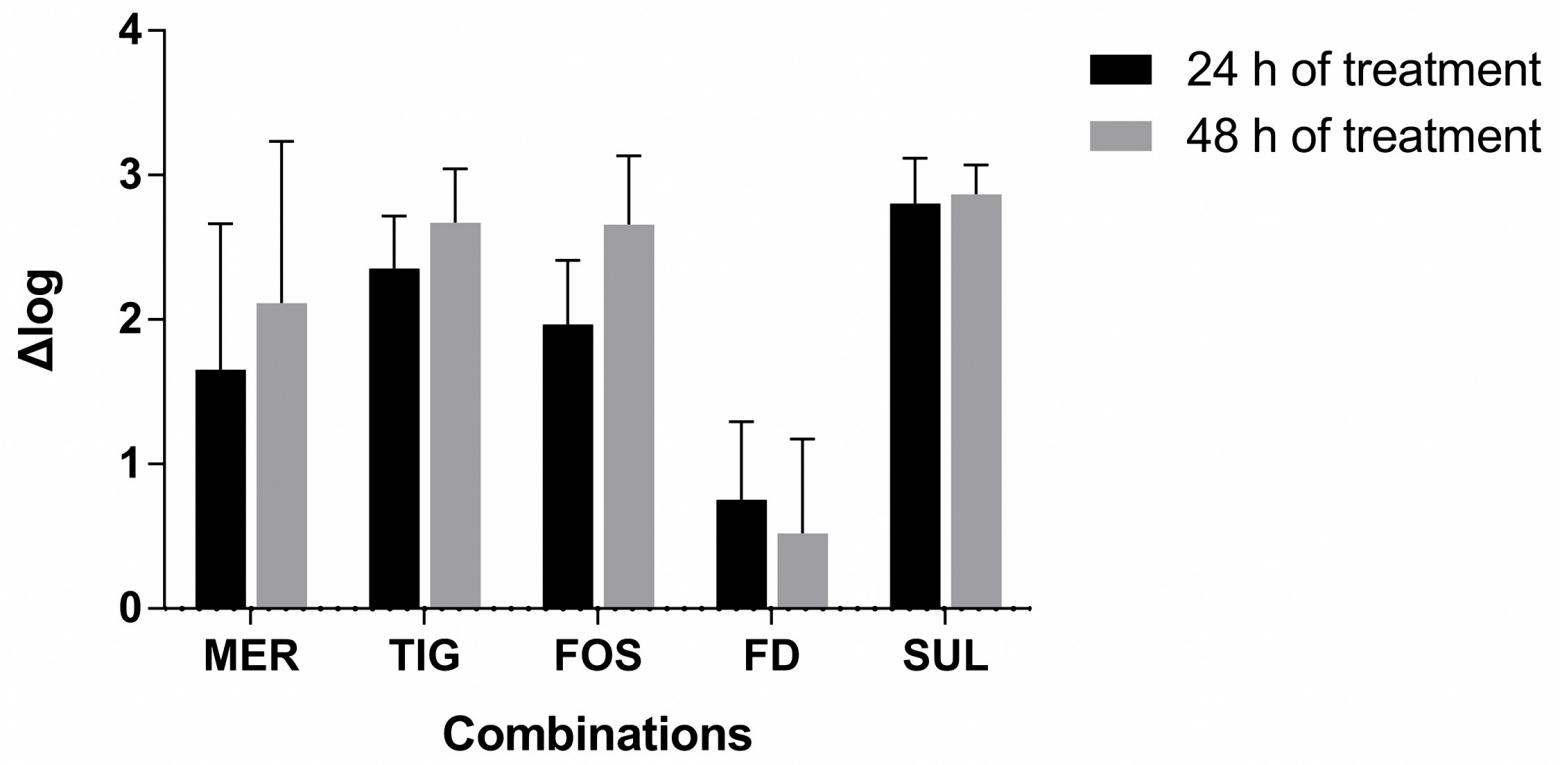
Comparison of the efficacies of colistin-rifampin combination vs other combinations after 24 and 48 h of treatment. CST, colistin; MER, meropenem; TIG, tigecycline; FOS, fosfomycin; FD, fusidic acid; RIF, rifampin; SUL, sulbactam. Δlog means the log_10_ recovered CFU of other combinations minus the log_10_ recovered CFU of colistin-rifampin combination.

As shown in Figs 2 and 3, colistin-fusidic acid and colistin-rifampin combinations were generally superior to other combinations. After a 48h treatment, a difference of >2 Δlog of bacterial counts was observed between the colistin-fusidic acid combination and the colistin-tigecycline, colistin-fosfomycin, or colistin-sulbactam combinations. When treating infections caused by strains with meropenem MICs < 64 mg/L, the combination of colistin with meropenem was superior to colistin with tigecycline, fosfomycin, or sulbactam, but were not inferior to colistin with fusidic acid after 24 h and 48 h of treatment (data not shown). After a 48h treatment, a difference of >2 Δlog of bacterial counts was observed between the colistin-rifampin combination and colistin treatment in combination with meropenem, tigecycline, fosfomycin, or sulbactam (Fig 3). Finally, there was no clear difference between the activities of the 2 most effective combinations (colistin plus fusidic acid versus colistin plus rifampin), with a < 1 Δlog of bacterial counts difference observed after both 24 h and 48 h of treatment for most strains.

## Discussion

XDR-AB strains threaten the successful treatment of serious infections in critical care patients worldwide [22]. Although several studies have evaluated the activities of diverse antimicrobial agents against XDR-AB, an optimal treatment scheme remains unclear. Some regimens involving colistin, tigecycline, and sulbactam (alone or in combination) may be efficacious in combatting XDR-AB infections [6], although limited *in vivo* data exists regarding the efficacies of these regimens.

In this study, we found that colistin, tigecycline, rifampin, and sulbactam monotherapy significantly decreased bacterial counts during murine thigh infections when compared with those observed in control mice receiving no treatment. Tigecycline and rifampin monotherapy displayed bactericidal activity in 50% (6/12) and 58.3% (7/12) of strains at 48 h post-treatment. Colistin monotherapy appeared to be the most effective monotherapy regimen, displaying bactericidal activity in 91.7% of strains after 48 h of treatment. This was in accordance with the findings of Pachon-Ibanez et al., whose pneumonia model also showed a bacterial reduction of approximately 3 log10 CFU/g in the colistin group relative to their control group [16]. However, results from a different study conducted by Montero et al. showed that colistin had the weakest antibacterial effect when compared with imipenem, sulbactam, tobramycin, and rifampin in a pneumonia model [23]. This discrepancy may be mainly explained by their low colistin doses used with an area under the curve (AUC) of 11.96 mg·h/L (Montero et al.), in contrast with the doses used in our study (the same as in the study by Pachon-Ibanez et al.), which resulted in an AUC of up to 26.42 mg·h/L, being similar to that in humans (23.43 mg·h/L). Moreover, the MICs of strains for imipenem, sulbactam, and rifampin in the study of Montero et al. were relatively lower than those of strains tested in our study.

Previous *in vitro* studies showed that the synergy rate of colistin combined with carbapenems against *A*. *baumannii* reached >80% [24]. However, the results of *in vivo* studies with the colistin-carbapenem combination were not synergistic as expected. A previous study by Song et al. showed that colistin in combination with imipenem did not significantly reduce bacterial loads in lungs after infection with an OXA-51-producing strain (MIC for imipenem: 64 mg/L) [25]. In our study, combination treatment with colistin and meropenem only showed synergistic effects in 41.7% of the test strains after 48 h of treatment (Table 4). We also noted that with strains having low meropenem MICs (≤ 32 mg/L), colistin-meropenem combination therapy displayed synergistic effects and was superior to colistin plus tigecycline, fosfomycin, or sulbactam. However, with strains having higher MICs (≥ 64 mg/L), no synergistic effects were found in this combination therapy. These results indicated that the colistin-carbapenem combination may not have advantages over monotherapy against infections caused by XDR-AB strains with high MICs for carbapenems.

Rifampin, which exhibited high MICs, was generally effective against XDR-AB strains, being comparable to colistin and tigecycline in our study. Previous *in vivo* studies using different animal models obtained similar outcomes [16, 23]. However, rifampin monotherapy is not recommended in clinical practice. Treatment with colistin plus rifampin yielded synergistic effects with all test strains at both 24 h and 48 h post-treatment. Compared with colistin combined with meropenem, tigecycline, fosfomycin, or sulbactam, administration of colistin with rifampin was more efficacious in treating XDR-AB infections. In a multicenter, open-label, randomized trial conducted with enrolled patients having life-threatening XDR-AB infections, including rifampin with colistin significantly increased the microbial-eradication rate (*P* = 0.034), but did not reduce the 30-day mortality [26]. Thus, the authors suggested that rifampin should not be routinely combined with colistin for treating severe XDR-AB infections at present. However, it should be noted that this study had important limitations, such as the lack of blinding of physicians to the intervention and the large number of patients who received active (tigecycline) or potentially synergistic (carbapenems) agents in the control group [27]. In the future, well-designed studies will be needed to clarify the role of colistin-rifampin combination therapy in treating XDR-AB infections.

The lack of effective, novel antibiotics against XDR-AB requires the use of unorthodox combinations of existing anti-microbial agents. A potent synergistic effect was observed *in vitro* between colistin and glycopeptides, daptomycin, or fusidic acid, which are only used against Gram-positive bacterial infections [28–31]. The effect is thought to be mediated via a permeabilizing effect of colistin on the outer membrane of *A*. *baumannii*, facilitating the entry of antibiotics with large molecules [32]. Using a simple *Galleria mellonella*-infection model, several studies have demonstrated that colistin combined with glycopeptides and daptomycin are highly active against *A*. *baumannii in vivo* [33–35]. Results from our study also revealed that the addition of fusidic acid significantly improved the efficacy of colistin, with a synergistic effect in 58.3% and 75.0% of the test strains after 24 h and 48 h of treatment. Colistin combined with fusidic acid was superior to the combination of colistin with meropenem, tigecycline, fosfomycin, or sulbactam. Therefore, the colistin-fusidic acid combination should be considered as a potential treatment for difficult-to-treat XDR-AB infections.

Although many *in vitro* studies have demonstrated that colistin combined with tigecycline, fosfomycin, or sulbactam displayed synergistic effects [8], the addition of these agents did not considerably improved the efficacy of colistin in the current study. Yilmaz et al. also showed that no statistically significant differences of bacterial counts in lung tissue were observed between colistin, tigecycline, and combination treatments against an XDR-AB strain, despite the synergy observed *in vitro* [36]. Previous data by Dinc et al. showed that the addition of sulbactam to colistin and tigecycline therapy had no significant effect on bacterial counts in an experimental model of carbapenem-resistant *A*. *baumannii* sepsis [21]. Thus, such colistin combinations might not be advantageous over colistin monotherapy for treating XDR-AB infections in clinical settings.

Our study has several limitations and should be interpreted with caution. First, although the strains used in this study were isolated from different patients in our hospital, this is a single-center study, and the strains segregated into only 4 groups, based on PFGE results. Second, we did not evaluate differences in dissemination of bacteria in the blood and other organs following treatment with different drug combinations. Third, because the thigh infection model is a local infection model, we could not compare mortality rates and other clinical data in addition to bacterial counts.

In summary, our *in vivo* findings suggest that colistin in combination with rifampin or fusidic acid is more efficacious in treating XDR-AB infections than are other combinations. Colistin-meropenem combination may be another appropriate option, in cases where the MIC is not higher than 32 mg/L. Further clinical studies are urgently needed to prove the relevance of these findings in humans.

## Supporting Information

S1 File Manuscript Database. (ZIP)
